# Immune Function and Muscle Adaptations to Resistance exercise in Older Adults: Study Protocol for a Randomized Controlled Trial of a Nutritional Supplement

**DOI:** 10.1186/s13063-015-0631-3

**Published:** 2015-03-27

**Authors:** Richard A Dennis, Usha Ponnappan, Ralph L Kodell, Kimberly K Garner, Christopher M Parkes, Melinda M Bopp, Kalpana P Padala, Charlotte A Peterson, Prasad R Padala, Dennis H Sullivan

**Affiliations:** Geriatric Research, Education and Clinical Center, Central Arkansas Veterans Healthcare System, 2200 Fort Roots Drive, 170/3 J, North Little Rock, AR 72114 USA; Donald W Reynolds Department of Geriatrics, University of Arkansas for Medical Sciences, 4301 West Markham, Little Rock, AR 72205 USA; Department of Microbiology and Immunology, University of Arkansas for Medical Sciences, 4301 West Markham, Little Rock, AR 72205 USA; Department of Biostatistics, University of Arkansas for Medical Sciences, 4301 West Markham, Little Rock, AR 72205 USA; College of Health Sciences, University of Kentucky, 900 South Limestone Street, Lexington, KY 40536 USA; Department of Psychiatry, University of Arkansas for Medical Sciences, 4301 West Markham, Little Rock, AR 72205 USA

**Keywords:** sarcopenia, skeletal muscle, aging, immune function, TDAP vaccination, resistance exercise, inflammation, growth factors, EAS Muscle Armor, Juven

## Abstract

**Background:**

Immune function may influence the ability of older adults to maintain or improve muscle mass, strength, and function during aging. Thus, nutritional supplementation that supports the immune system could complement resistance exercise as an intervention for age-associated muscle loss. The current study will determine the relationship between immune function and exercise training outcomes for older adults who consume a nutritional supplement or placebo during resistance training and post-training follow-up. The supplement was chosen due to evidence suggesting its ingredients [arginine (Arg), glutamine (Gln), and β-hydroxy β-methylbutyrate (HMB)] can improve immune function, promote muscle growth, and counteract muscle loss.

**Methods/design:**

Veterans (age 60 to 80 yrs, N = 50) of the United States military will participate in a randomized double-blind placebo-controlled trial of consumption of a nutritional supplement or placebo during completion of three study objectives: 1) determine if 2 weeks of supplementation improve immune function measured as the response to vaccination and systemic and cellular responses to acute resistance exercise; 2) determine if supplementation during 36 sessions of resistance training boosts gains in muscle size, strength, and function; and 3) determine if continued supplementation for 26 weeks post-training promotes retention of training-induced gains in muscle size, strength, and function. Analyses of the results for these objectives will determine the relationship between immune function and the training outcomes. Participants will undergo nine blood draws and five muscle (vastus lateralis) biopsies so that the effects of the supplement on immune function and the systemic and cellular responses to exercise can be measured.

**Discussion:**

Exercise has known effects on immune function. However, the study will attempt to modulate immune function using a nutritional supplement and determine the effects on training outcomes. The study will also examine post-training benefit retention, an important issue for older adults, usually omitted from exercise studies. The study will potentially advance our understanding of the mechanisms of muscle gain and loss in older adults, but more importantly, a nutritional intervention will be evaluated as a complement to exercise for supporting muscle health during aging.

**Trial Registration:**

Clinicaltrials.gov identifier: NCT02261961, registration date 10 June 2014, recruitment active.

## Background

**Figure 1:**
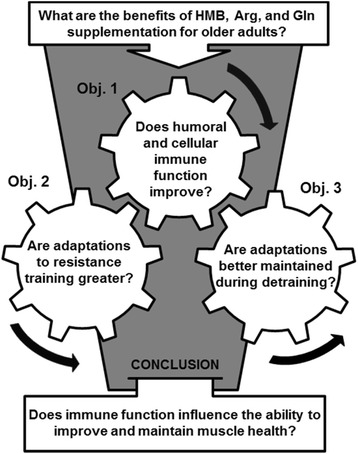
**Study objectives for a randomized trial of a nutritional supplement designed to determine if immune function influences the ability of older adults to improve muscle mass, strength, function, and cellular adaptation to resistance exercise training.**

Effective strategies for rehabilitation are critical to the health of older adults since age-associated muscle loss decreases functional ability and increases risk for falls and fractures, disability, and mortality [1,2]. Resistance exercise training can be an effective intervention for improving muscle mass, strength, and function in older adults, but the magnitude of benefit varies considerably among individuals [3,4]. This suggests that older adults with less adaptive potential will have greater muscle loss due to aging and that their rehabilitation will be less effective. Thus, a better understanding of the effects of aging on the cellular and biochemical mechanisms controlling muscle health may be needed to design interventions (for example, nutritional supplementation) that complement resistance training. Specifically, this protocol will determine whether immune function influences muscle adaptation to resistance exercise and whether the influence is affected by a nutritional supplement (Arg, Gln, and HMB), whose ingredients appear capable of improving immune function, promoting muscle growth, and counteracting muscle loss [5]. A randomized controlled trial is being conducted in which older adults consume a nutritional supplement or placebo during a resistance training program and post-training period in order to determine if the supplement improves immune function, boosts muscle adaptation to training, and supports post-training retention of the exercise-derived benefits (Figure 1).

The bodily systems are often co-dependent during health and dysfunction, but the role of immune function in maintaining or improving muscle health for older adults is unknown. The study will investigate the effects of nutritional supplementation on variables for humoral, innate, and adaptive immune functions and determine their relationship to exercise training outcomes. With aging, the antibody response to vaccination is weaker and wanes sooner over time, meaning that older adults may not possess protective antibody titers [6]. T-cells and monocytes have a diminished ability to proliferate and CD4+ helper t-cells, the CD8+ suppressor t-cells, and other t-cell subsets become imbalanced [7]. These changes increase susceptibility to infectious disease but may also indicate that other systemic problems exist that may specifically affect muscle. Acute increases in plasma pro- and anti-inflammatory cytokines after acute exercise are thought to initiate muscle repair of damage [8,9], and repetition of this immune response through training is thought to be involved in muscle adaptation [10]. However, with aging, acute responsiveness gives way to chronic inflammation that may be detrimental to muscle. In rats, cytokine infusion suppresses protein synthesis and induces muscle catabolism [11]. In humans, elevated cytokines are associated with sarcopenia and functional decline [12]. Thus, systemic differences in immune parameters (for example, response to vaccine, t-cell phenotype, or cytokine levels) between older adults may reflect the individual ability to maintain or improve muscle health despite aging.

Specific cells of the immune system, that is, macrophages, appear to play an important role in muscle hypertrophy [13]. Macrophages are thought to transition between three functional phases in response to unaccustomed exercise [14]. Phase 1 is an inflammatory response mediated by systemic and local cytokines, such as IL1β and TNFα, which initiate muscle repair. Macrophages, termed M1 macrophages, produce these pro-inflammatory cytokines and chemokines to recruit additional macrophages to remove cellular debris from damaged myofibers [15]. After this initial cleanup, M2 macrophages function in Phase 2, which includes the downregulation of inflammation by anti-inflammatory cytokines, such as IL-1RA and IL10, so that secondary damage does not occur. This allows muscle tissue to become conducive to growth [16]. In the regenerative Phase 3, macrophage arginine metabolism increases and its byproducts promote cell growth [17]. Growth factors such as IGF1 are produced to further stimulate myogenesis [18]. Satellite cells proliferate and fuse with existing fibers so that the added myonuclei can support an increase in fiber size [19]. However, muscle injury is not necessary for macrophage stimulation [20]. Thus, in the absence of damage, or as muscle adapts to training, macrophages may be predominantly dedicated to producing growth factors required for muscle maintenance or hypertrophy.

**Figure 2:**
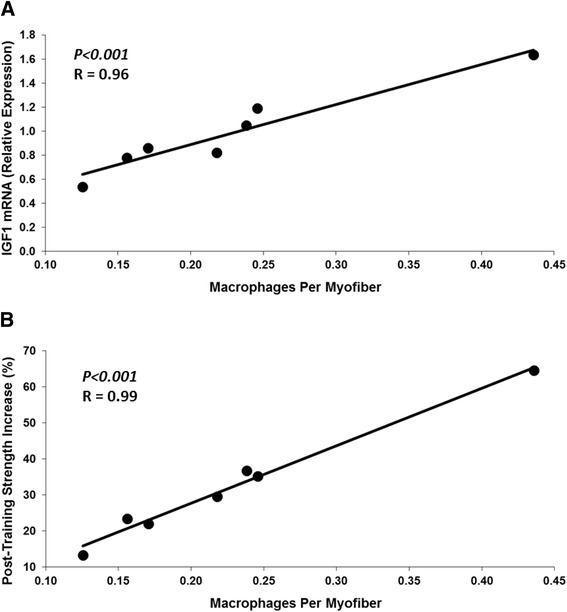
**Relationships between pretraining muscle molecular and cellular measures and percent strength gain after 12 weeks of high-intensity progressive resistance training of the thigh muscles for older adults (N = 7, Age 69 ± 6 yrs). (A)**. Pearson correlation between the number of macrophages and IGF1 mRNA. The number of macrophages staining positive for CD163 by immunohistochemistry were counted per myofiber. IGF1 mRNA was measured using quantitative real-time reverse transcriptase polymerase chain reaction. **(B)**. Pearson correlation between the number of muscle macrophages and strength gain for knee extension. One outlier was excluded from these analyses due to having macrophage levels that were fourfold higher than the group average.

Evidence from animal and human studies suggests that the immune system and macrophages influence muscle mass and strength during health and aging. In animals, experimental reduction of muscle macrophage content decreases the ability of muscle to hypertrophy and recover from atrophy [21-23]. The effects were related to decreased macrophage provision of the growth factor IGF1 and decreased satellite cell differentiation, myonuclear addition, and muscle fiber growth [21,23]. In humans, muscle mass and strength gain are also associated with increases in muscle satellite cells, fiber nuclei, and fiber size [24]; and these adaptations to exercise are diminished in older adults [25]. Inflammation may have contributed to those findings. In a training study of older adults, individuals consuming anti-inflammatory drugs gained significantly more muscle mass and strength than those consuming placebo [26]. Our studies also indicate that immune function influences the response to exercise. The muscle cross-sectional area and strength gained by older adults from resistance training showed a strong positive correlation with muscle expression of specific pro- and anti-inflammatory cytokines and growth factors including IGF1 [4]. Muscle macrophage content pre-training in those older adults was also strongly correlated with pretraining growth factor levels (R = 0.96, *P* <0.001) and post-training strength gain (R = 0.99, *P* <0.001) (Figure 2). These results suggest that macrophages may be regulated by cytokines during exercise and provide growth factors that promote the adaptation of muscle cells and fibers, which leads to mass and strength gains. Thus, consumption of a nutritional supplement that either dampens a negative or boosts a positive effect of inflammation and immune function could benefit older adults during exercise training.

**Figure 3:**
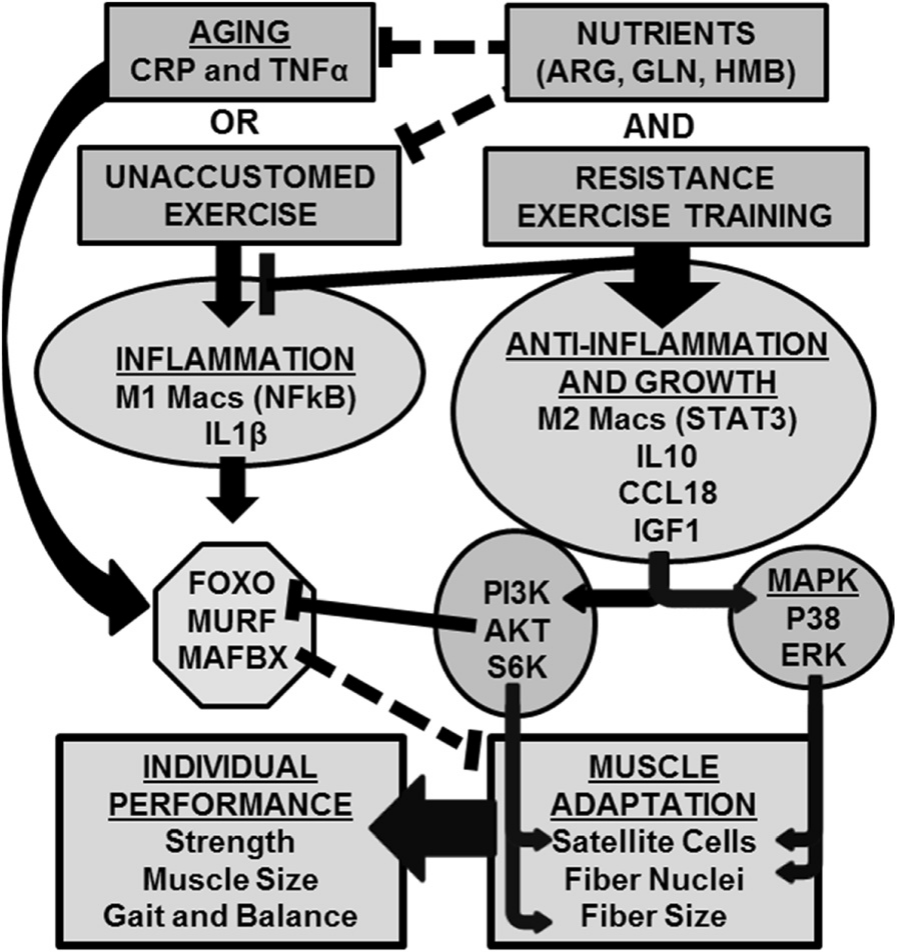
**Proposed model for how a nutritional supplement promotes a shift in immune system function in older adults from a pro-inflammatory state (both at rest and in response to a bout of exercise) towards a state in which inflammation is dampened so that muscle growth in response to exercise training is supported.** The supplement is proposed to augment muscle adaptation and performance gains from resistance training by altering macrophage and muscle cell phenotype through specific cytokines, growth factors, and signaling pathways.

Three signaling pathways may be involved in the regulation of macrophage function, production of cytokines and growth factors and their effects on muscle cells. M1 macrophages are regulated primarily by NFkB in response to systemic pro-inflammatory cytokines, such as TNFα and IL1β [27]. M1 macrophages then may be responsible for production of cytokines, which block FOXO phosphorylation and lead to increased transcription of the ubiquitin ligases, MuRF and MAFbx, which control protein degradation and can lead to muscle atrophy [28]. Macrophage conversion to M2 function is driven by and results in production of anti-inflammatory cytokines, such as IL10 and CCL18, through the activation of STAT3 [27]. These macrophages strongly support tissue growth, as was originally characterized due to the poor prognosis associated with their presence in tumors. M2 macrophages may be a source of IGF1, which promotes muscle health by blocking the FOXO pathway and stimulating the PI3K and MAPK signaling cascades. Activation or blockage of factors within these pathways results in obvious effects on muscle. For example, constitutive AKT signaling results in muscle growth; whereas deletion of the S6K results in muscle atrophy [29]. Examination of these pathways in human muscle is limited to date, and further investigation is warranted. A model for how these signaling pathways, macrophage function, and cytokines and growth factors hypothetically integrate to control muscle health (that is, adaptation and performance) in the context of aging, nutritional supplementation, and exercise is presented in Figure 3.

A nutritional supplement that affects immune function may improve the ability for older adults to reap and retain the benefits of resistance training. The supplement ingredients (Arg, Gln, and HMB) are used for nutritional support during conditions associated with muscle wasting or requiring tissue regeneration [5]. These ingredients have shown success in slowing cachexia and increasing fat-free body mass in both cancer and AIDS patients [30,31]. The ingredients also increased fat-free mass, strength, and muscle function after 12 weeks of consumption by elderly women. They are also commonly used to increase muscle mass and strength in combination with resistance exercise for sports performance [5,32,33]. Despite this success, little is known about the mechanism of action. HMB may work by altering protein synthesis, but it also enhances humoral immune function at least in animals [34-36]. Glutamine and arginine are considered immuno-nutrients. They affect immune cells including lymphocytes, T-cells, and macrophages by altering cell phenotype, proliferation, and production of pro- and anti-inflammatory cytokines [37-40]. Thus, the supplement or its ingredients are used to support muscle health in the extreme conditions of cachexia and sports performance, and numerous effects on immune function have been identified. For these reasons, conduct of a randomized controlled trial of this particular supplement offers an opportunity for a mechanistic investigation of the relationship between immune function and muscle adaptation to exercise in older adults.

### Study objectives

The objectives of this trial are as follows:

1. Determine the effects of nutritional supplement consumption by older adults on the humoral response to vaccination and specific cellular immune responses to an acute resistance exercise stimulus.
2. Determine if nutritional supplement consumption affects the adaptive responses to resistance exercise training at the whole body or cellular level.
3. Determine if nutritional supplement consumption affects the retention of benefits of exercise during post-training follow-up.

## Methods/design

### Human subject participation

#### Trial summary

**Figure 4:**
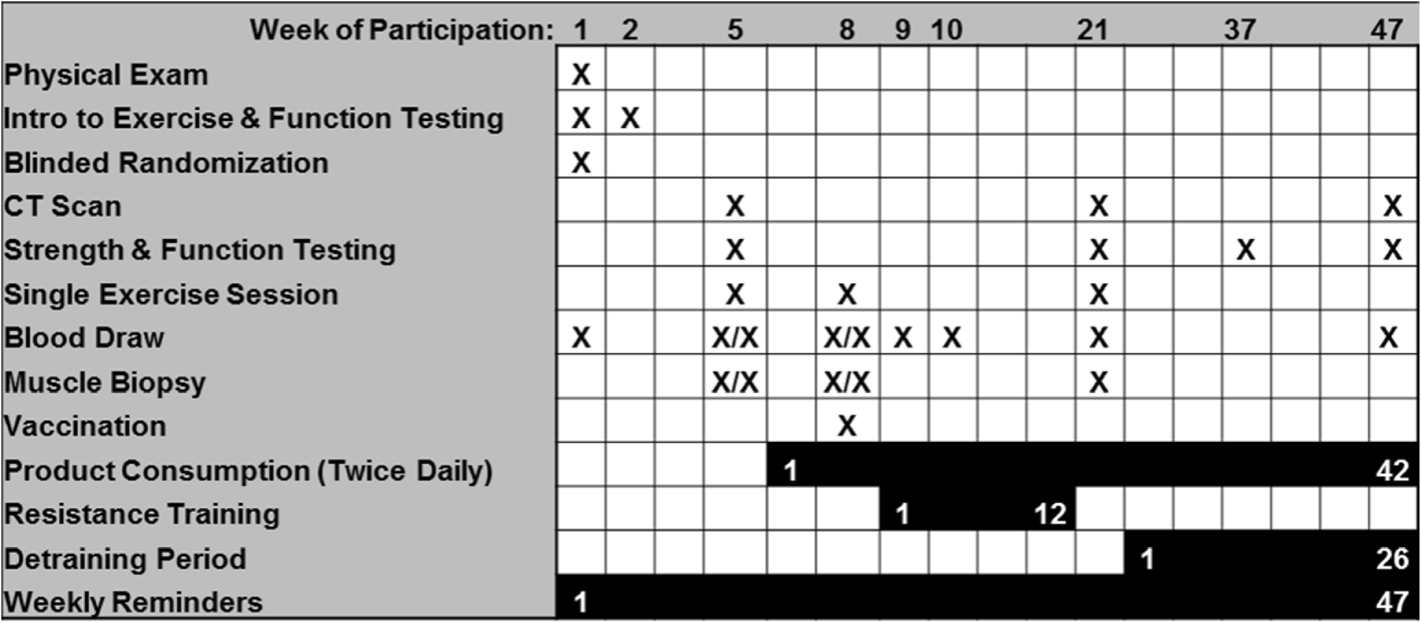
**Timeline for individual subject participation.** Each individual will participate in the study for approximately one year. Participation will include consumption of a nutritional supplement or placebo twice daily for 42 weeks. During this time, subjects will also complete 12 weeks of resistance training followed by 26 weeks of post-training follow-up (that is, a detraining period). The particular procedures and assessments involved during each week of participation are listed. X/X denotes assessment of response to a single bout of exercise by collecting specimens before and after exercise for blood (1 hour) and muscle (72 hours). The scheduling timeline is approximate and can be extended based on the scheduling needs of the subjects and study staff.

Subjects will participate in a randomized double-blind placebo-controlled trial of a nutritional supplement (Figure 4). Individual participation will optimally last 47 weeks. This time will include periods for introduction to exercise and testing of muscle function (2 weeks), supplementation prior to training (2 weeks), supplementation plus supervised resistance training (12 weeks), and supplementation during post-training follow-up (that is, detraining, 26 weeks). Subjects will undergo five muscle biopsies and nine blood draws total. The effects of the supplement or placebo on participant responses to a single bout of exercise, vaccination (tetanus, diphtheria, and pertussis (TDAP)), exercise training, and detraining will be measured. The study outcomes are listed in Table 1. Participation will require approximately 55 study visits. Subjects will be compensated for their time via gift cards redeemable at a local grocery store in the amount of $20 per visit plus bonuses of $100 for completing the resistance training and $300 for completing the entire study. The study has been approved as required by all entities of the Central Arkansas Veterans Healthcare System (CAVHS) including the Research and Development Committee, CAVHS Institutional Review Board (IRB protocol 608119), Subcommittee for Research Safety, Privacy and Information Security Officers, and Public Affairs Office. The study is being conducted in the Geriatrics Research, Education and Clinical Center of a Veterans Affairs Medical Center.

**Table 1:** **Study outcomes for immune responses to various stimuli and for muscle adaptation to training**

**Immune function**	**Muscle adaptation**
**Blood measures**	**Whole body measures**
Antibody response to TDAP vaccine^1^	1-Repetition maximum strength^5^
Pro- and anti-inflammatory T-Cell population balance^2^	Thigh cross-sectional area (CT scan)
Pro- and anti-inflammatory cytokine/chemokine levels^2^	6-minute walk and gait speed tests
Proliferation of peripheral blood mononuclear cells^3^	Berg Balance Scale
C-Reactive Protein^5^	Timed Up and Go test
**Muscle tissue measures**	**Muscle tissue measures**
Change in CD163+ Macrophages^2, 5^	Change in satellite cell numbers
Changes in pro- and anti-inflammatory cytokines^2^	Change in fiber size
Growth factor levels^4^	Nuclear addition to muscle fibers
NFkB, PI3 Kinase, FOXO, and STAT3 signaling pathways^2^	

^1^Humoral response to the tetanus, diphtheria, and pertussis (TDAP) vaccine; ^2^Response to an unaccustomed bout of exercise; ^3^ *In vitro* stimulation assay; ^4^Resting measure; ^5^Primary outcome measures used in power calculations while other outcomes are secondary.

#### Recruitment and enrollment

Veterans (age 60 to 80 yrs, N = 50) of the United States military are being recruited from the local community and enrolled by study staff to participate in the 4-year study. The county of the study site and the surrounding counties contain approximately 36,000 veterans who meet the age requirements for the study. An estimated 92% are white, 6% are black, and 3% are female [41]. Ethnic and racial groups are expected to enroll at least in proportion to their representation in the community. Vulnerable populations will not be targeted. Recruitment will utilize flyers posted in the VA hospital, clinician referral, and newspaper advertising. Recruits will be pre-screened by phone interview and review of their electronic medical record. Recruit permission and the necessary waivers of HIPAA and written consent will be used for this process. Individuals appearing eligible will provide written consent at their first research visit. Subjects will undergo a medical history and physical exam performed by the study physician, including electrocardiogram during resistance exercise. The physician will determine eligibility based on the American College of Sports Medicine guidelines for resistance exercise and the exclusion criteria related to specific study procedures (Table 2) [42].

**Table 2:** **Study inclusion and exclusion criteria**

**Inclusion criteria**	
• U.S. Military Veteran	• Age 60 to 80 years
• Nonsmoker of tobacco products	• Body Mass Index of 18.5 - 29.9 kg/m^2^
**Exclusion criteria**	
• Unstable angina	• Uncontrolled diabetes mellitus (HbA1C > 10)
• Allergic to lidocaine	• Problems walking or exercising with both legs
• Allergic to vaccination	• Metastatic cancer or undergoing chemotherapy
• Allergic to latex or tape	• Hernia that causes pain during physical activity
• Seizure in past 3 months	• Tetanus or TDAP vaccine in previous two years
• Smokes tobacco products	• Certain steroid or androgen use in past 3 months
• Other physician judgment	• Cerebral aneurysm or intracranial bleed in past year
• Systemic bacterial infection	• Unwilling to halt new use of nutritional supplements
• Active oral or genital herpes	• End-stage congestive heart failure (NYHA Stage IV)
• Bleeding or clotting disorders	• Unstable abdominal or thoracic aortic aneurysm (>4 cm)
• Encephalopathy in past 7 days	• Myocardial infarction or cardiac surgery in past 3 months
• Untreated severe aortic stenosis	• Pains, tightness, pressure in chest during physical activity
• Renal disease requiring dialysis	• Significantly abnormal blood tests (CBC or PT/PTT/INR)
• Uncontrolled asthma or allergies	• Bone fractures in the pelvis, legs, or feet in the last 3 months
• Significant problem with fainting	• Taking prescription anti-coagulants (that is, heparin, warfarin, *etcetera*)
• Current use of appetite stimulants	• Current treatment for mania or bipolar disorder or taking lithium
• History of peripheral artery disease	• Pulmonary embolism or deep venous thrombosis in past 3 months
• Active suicidality or suicidal ideation	• Taking lactulose, nitrates plus hypertension medications or Viagra
• Significant problems with chronic pain	• Uncontrolled hypertension or hypotension (>160/100, <100 systolic)
• Unwilling to maintain current normal diet	• Acute retinal hemorrhage or ophthalmologic surgery in past 3 months
• Diagnosis of a significant cognitive deficit	• Unwilling to halt concurrent use of amino acid or protein supplements
• Guillain-Barre Syndrome in past 3 months	• Proliferative diabetic retinopathy or severe nonproliferative retinopathy
• Uncontrolled malignant cardiac arrhythmia	• Participated in a weight-lifting program targeting the thighs in last 3 months
• Liver cirrhosis or other severe liver disease	• Currently participating in any other research study involving an intervention
• Taking aspirin in any form and unable/unwilling to discontinue for 10 days prior to muscle biopsy	
• Taking any non-aspirin non-steroidal anti-inflammatory drug and unable or unwilling to discontinue use for 3 days prior to the muscle biopsy procedure	
• Taking Fish Oil, Gingko, Garlic, Saw Palmetto, Turmeric, or Vitamin E and unable or unwilling to discontinue use for 10 days prior to the muscle biopsy procedure	

#### Randomization

Subjects will be randomized to parallel groups to consume either nutritional supplement or placebo. The hospital research pharmacist will perform the double-blind randomization using variable block sizes with an allocation ratio of 1:1. The randomization scheme was generated using the website http://www.randomization.com. Assignments will be unknown to investigators and subjects. The study will enroll 50 subjects total to allow for up to 20% dropout to have at least 40 complete the protocol. The hospital pharmacy will dispense supplement and placebo in blinded packaging. The protocol appointed a study monitor (specific un-blinded member of the study team) to review and be involved in pharmacy and subject compliance but not involved in subject assessments or specimen analysis.

#### Supplementation

The ingredients of the nutritional supplement (Muscle Armor, Abbott Laboratories, Columbus, OH) and placebo (Kool-Aid, Kraft Foods Group, Northfield, IL) are listed in Table 3. Subjects in the supplement group will consume orange-flavored supplement according to the manufacturer’s directions: one serving (approximately 30 g), twice daily (optimally morning and evening), mixed with 12 oz of water. Subjects in the placebo group will similarly consume approximately 13 g of orange-flavored placebo mixed with 12 ounces of water twice daily. Supplementation will begin the day of the second muscle biopsy and continue for the remainder of the participant’s study involvement. Compliance will be monitored by subject self-report on a dosing calendar as well as by the study monitor calculating the weight of product consumed. Products will be dispensed in blinded sealed canisters containing 14 doses. Subjects will return any unused product. The monitor will review dosing and consumption for compliance with the subject approximately every 4 weeks or more often as needed. Subject dietary data will not be collected but to be involved subjects must agree to maintain their normal diet during participation.

**Table 3:** **Comparison of nutrient information between nutritional supplement and placebo mixed in 12 oz water**

**Supplement**	**Per serving**		**Placebo**	**Per serving**	
**AMINO ACIDS**			**AMINO ACIDS**		
L-ARGININE	7	g	L-ARGININE	0	g
L-GLUTAMINE	7	g	L-GLUTAMINE	0	g
TAURINE	3	g	TAURINE	0	g
**CARBOHYDRATE**	10	g	**CARBOHYDRATE**	12	g
SUGARS	5	g	SUGARS	12	g
**MINERALS**			**MINERALS**		
CALCIUM	200	mg	CALCIUM	0	mg
Calcium β-hydroxy-β-methylbutyrate	1.5	g	Calcium β-hydroxy-β-methylbutyrate	0	g
VITAMIN C	0	mg	VITAMIN C	7	mg
**ENERGY**	110	kcal	**ENERGY**	46	kcal

#### Vaccination

The TDAP vaccine (Adacel, Sanofi Pasteur, Swiftwater, PA) will be administered by a study physician or nurse to each subject after 2 weeks of supplementation (day of third muscle biopsy) (Figure 4). Blood will be collected at baseline and 1 and 2-weeks post-immunization to determine the effects of the supplement on the humoral response. Two post-vaccination time-points were chosen in case the response is delayed in older adults relative to the response in younger adults [43]. If needed to accommodate scheduling, the supplementation period may be extended and the two post-vaccination blood draws may be delayed by one day if necessary.

#### Resistance exercise

The study will use a resistance exercise routine that we have previously used to elicit cellular and molecular responses in muscle associated with immune function and to improve muscle mass and strength of the thigh for older adults [4,9,14,44]. The resistance exercise program will use three exercises to strengthen the thigh muscles, including bilateral knee extension, knee curl, and leg press. Exercises will be completed on air-driven exercise equipment (Keiser, Fresno, CA) in a seated position and always supervised by a study team member. Strength testing will utilize the 1-repetition maximum (1RM) test [45]. A brief warm-up period will include 10 minutes of mild aerobic activity on a cycle ergometer. For each exercise, approximately six repetitions of lifting warm up will be completed at a ‘somewhat heavy’ (that is, approximately 65% 1RM) load. The load will then be gradually (approximately 5 to 10%) increased such that the maximum capability for lifting is reached in approximately ten total repetitions. Subjects will rest 60 seconds between repetitions after the warm up. Subjects rest 5 minutes prior to the next test completed in the order of press, curl, and extension. Subjects will complete one introduction to the exercise session and undergo strength testing twice during the first 2 weeks of participation. Subjects will then have at least 2 weeks of rest prior to the unaccustomed exercise sessions. The purpose of this rest period is to mitigate any possible effects on the muscle tissue analysis.

#### Unaccustomed exercise sessions

Prior to exercise training, the study will measure the response to single bouts of the training exercises before and after two weeks of nutritional supplementation (Figure 4). The unaccustomed exercise session will be the same as each session completed for exercise training as described below. The response to exercise will be measured using both muscle and blood. The pre-exercise biopsy time points will be prior to exercise, possibly the same day or a different day, based on scheduling and/or the judgment of the study physician. The post-exercise time points for collection are 72 hrs for muscle and 1 hr for blood. Our studies have previously shown that 72 hrs is better than 24 hours for measuring the immune variables of interest in muscle [9]. The 1 hr time point for blood collection is based on other studies that have determined the effects of exercise on systemic measures of immune function [8,46].

#### Exercise training sessions

The training program will have subjects perform supervised exercise for approximately an hour three times per week on nonconsecutive days. The program consists of 36 exercise sessions that will optimally take 12 weeks though the total time will likely be longer due to scheduling based on the needs of the subject or the study team. The exercise load will be based on the subject 1RM. After a light cycling warm up, subjects will perform four sets of each exercise at loads of 60, 70, 75, and 80% of their measured capability. Strength is retested at every sixth session to account for strength gain in maintaining the work load. On days of testing, the strength test replaces the first of the four exercise sets. Strength is also tested twice prior to training, at the final training bout, and twice during the post-training follow-up period. Training sessions consist of three sets of ten repetitions for each exercise and a fourth set completed at voluntary failure to complete a repetition. Subjects will rest approximately 2 minutes between each set and approximately 5 minutes between exercises. Exceptions to the plan for each subject to complete 36 exercise training sessions may be necessary on a case-by-case basis in order to ensure that the final muscle biopsy of the protocol, needed for assessment of muscle adaptation, occurs 72 hours (3 days) after the final exercise training session. After each exercise session or as needed during exercise, subjects will be guided through stretching of the quadriceps, hamstrings, and other affected muscles.

#### Post-training follow-up

The subjects will remain in the study after completion of the training program for 26 additional weeks. The retention of muscle mass, strength, and function will be examined relative to continued daily consumption of supplement or placebo (Figure 4). Subjects will be required to refrain from strength training of the thighs (that is, weight lifting, body weight exercise, and elastic bands) during this period. It is acceptable for subjects to participate in other forms of exercise such as walking. Compliance with these and study dietary requirements will be monitored by subject query at monthly visits during the follow-up period. These visits are also required for dispensing of product from the pharmacy and monitoring of consumption compliance.

#### Functional assessments

Functional assessment data will be collected at four times during the protocol: pre-training, post-training, during post-training follow-up, and at end of the post-training follow-up (Figure 4). Four assessments of muscle function will be performed. The Berg Balance Scale will be used to detect balance impairments in older adults while the individual is static or performing various movements [47]. The Six Minute Walk Test will be used to measure mobility performance [48]. The subjects will perform a supervised timed walk on a 200-foot track at a pace without encouragement and that allows speech without shortness of breath. The Gait Speed will be measured as habitual walking speed [49]. The subjects will walk at their normal preferred pace for 14 meters though only the middle 10 meters are timed. The result is an average of three replicates. Subjects will also complete the Timed Up and Go test. The test is indicative of balance and mobility by a measuring the time required for the subject to rise from a chair, walk 3 meters, turn around, walk back to the chair, and sit down [49].

#### Muscle cross-sectional area

Muscle size will be determined by the hospital imaging service using a CT scan of the mid-thigh of the dominant leg at three time points during the protocol: pre-training, post-training, and at end of the post-training follow-up (Figure 4). Subjects will rest in a supine position for 30 minutes prior being precisely positioned in the scanner. The CT image will then be taken at the midpoint between the inguinal fold and proximal pole of the patella, along the femur. A blinded analysis of the scans will be completed using Slice-O-Matic software (Montreal, Canada) to discern between Hounsfield units for bone (+1000), fat (-190 to -30), and normal (40 to 100) and low (0 to 30) density muscle.

#### Muscle biopsy and blood draws

Subjects will undergo five muscle biopsies and nine blood draws in order to assess the effects of nutritional supplementation on immune function and cellular measures of muscle adaptation (Figure 4 and Table 1), though blood will also be used to assess subject eligibility and safety. Muscle tissue will be obtained from each subject by needle biopsy of the vastus lateralis (outer thigh) by a study physician. The procedure is performed after local anesthetic (bicarbonate buffered lidocaine) is used to numb the site. A small incision will be made in the skin and a Bergstrom needle will be inserted briefly into the muscle so that suction and closing of the needle can be used to obtain 100 to 200 mg of muscle. Direct pressure will be applied to stop the bleeding and the wound will be closed and covered with a pressure bandage. Pre-exercise biopsies will be taken from the dominant leg while post-exercise biopsies will be taken from the nondominant leg. Blood draws will be performed by a study phlebotomist or unit nurse. The blood will most likely be drawn from the antecubital vein (≤60 ml or 2 oz). Fasting is not a requirement.

#### Data safety and monitoring plan

The following plan has been adapted from ‘Guidelines for Data and Safety Monitoring for Clinical Trials Not Requiring Traditional Data Monitoring Committees’ [50]. The study has eligibility criteria that have been precisely defined to ensure enrollment of subjects who possess an appropriate risk/benefit ratio (Table 2). Safety monitoring will be performed from enrollment until at least 30 days postparticipation for each subject. Monitoring occurs by query of the patient medical record and subject self-report or exam at each research visit, as well as during telephone contacts. Expected adverse events are listed in the informed consent document and will be monitored for, as will serious adverse events and unexpected adverse events possibly related to participation. Appropriate action relative to the severity of the event will be taken by the study team immediately when an event is identified. This may range from documentation by the coordinator (for example, mild muscle soreness) to safety examination by the study physician (for example, elevated blood pressure after exercise). The study procedures (for example, exercise or supplementation) will be halted at any time a safety concern is identified. Halted procedures will not resume until the concern has been alleviated. If concerns cannot be alleviated, then the study physicians may rule to withdraw the subject, modify the protocol, suspend enrollment, or halt the study. This is a relatively small single-site study, which allows decisions to be made in real-time as problems occur, rather than after aggregate data collection and interim analysis. Adverse events will be reported in compliance with IRB policy.

### Research laboratory analyses

#### Blood measures

Plasma and monocytes derived from blood draws at the time points listed in the Statistical Plan section will be used to measure four types of variables related to immune function (Table 1):

1. The responses to vaccination against tetanus, diphtheria, and pertussis will be assessed using enzyme-linked immunosorbent assays to measure antibody titers to the vaccine antigens (IBL International, Toronto, Ontario).
2. The study will determine if 2 weeks of supplementation increases the ratio of naïve helper (CD4+) to cytotoxic (CD8+) T-cells at rest or in response to acute exercise. The effects on other T-reg (T-regulatory) or Th17 T-cell subsets may also be examined. T-cell subset analysis will be carried out by flow cytometry using fluorochrome-linked antibodies specific to each marker (BD Biosciences, San Jose, CA).
3. The levels of pro- and anti-inflammatory cytokines and chemokines will be measured in plasma. Examples include Th1 cytokines (IFN-γ, TNF-α, IL-2, and IL-17) and Th2 cytokines (IL-10 and IL-4). Cytokines and chemokines will be measured using the multiplex Luminex array system (Bio-Rad, Hercules, CA, USA) and confirmed using high sensitivity enzyme-linked immunosorbent assays (R & D Systems, Minneapolis, MN, USA). C-reactive protein levels will also be measured by the hospital clinical laboratory.
4. The study will also evaluate immune cell proliferative ability for each individual. The study will determine if 2 weeks of supplementation increases the *in vitro* proliferative capacity of peripheral blood mononuclear cells at either baseline or after acute resistance exercise. Proliferation will be measured by flow cytometry based on uptake of a fluorescent dye (carboxyfluorescein succinimidyl ester) after treatment with phorbol ester and ionomysin, a calcium ionophore [51].

#### Muscle measures

Muscle tissue collected by biopsy at the time points listed in Figure 4 will be used to measure four types of variables related to immune function and muscle adaptation (Table 1):

1. The study will determine if 2 weeks of supplementation increases the number of macrophages present in muscle at baseline or in response to acute resistance exercise. Macrophages will be counted in muscle cross-section by immunohistochemistry using antibodies to detect the CD163 marker (Cell Sciences, Canton, MA). Additional markers (CD68 and CD206) will be used to confirm the specificity of the CD163 marker to macrophages.
2. Muscle levels of cytokines and growth factors will be assessed. The study will determine if 2 weeks of supplementation decreases baseline cytokine levels and increases the response to acute exercise. The study will also determine if supplementation increases growth factor levels at baseline and in response to acute exercise. IL1β, IL10, and IGF1 are of primary interest, though other cytokines and growth and remodeling factors including MuRF and MAFbx will also be measured. Gene expression will be measured at the mRNA level using total RNA extracted from whole muscle by quantitative real-time PCR using SYBR green chemistry and standard curves [4,9,44].
3. The study will evaluate signaling proteins that may be involved in the regulation of muscle phenotype. The study will determine if 2 weeks of supplementation decreases NFkB signaling and increases PI3K signaling at baseline and in response to exercise. NFkB activity will be assessed by electrophoresis mobility shift assays to detect muscle nuclear protein binding to the NFkB DNA site [52]. PI3K activity will be assessed by Western Blot based phosphorylation assays (Millipore, Billerica, MA). Other signaling proteins of interest include FOXO1, STAT3, AKT, S6K, and ERK1 and 2. Matched antibody pairs are available to measure the ratio of these phosphorylated to un-phosphorylated proteins by Western Blot (Cell Signaling Technology, Boston, MA).
4. Muscle adaptation to training will be measured at the cellular levels by analyses of satellite cells and myofibers. The study will determine if supplementation during resistance training increases the number of satellite cells and myonuclei per myofiber in muscle compared to training without supplementation. Satellite cells (Pax7+, CD56+, DAPI+) and myonuclei (Dystrophin plus DAPI staining) will be counted using established staining and immunohistochemistry methods [24]. Changes in myofiber size in response to training and supplementation will also be measured microscopically, as the average percent increase in fiber cross-sectional area from baseline to post-training.

### Statistical plan

#### Study power and hypothesis

The sample size (N = 50) will allow for 20% drop-out, while providing 80% power for the study objectives to detect mean differences between groups of 0.8 to 0.85 SD units at a 5% level of significance. Power calculations were based on using a one-sided *t*-test to compare independent groups using data from our work and others [14,26,53] though non-parametric tests or transformations will be used as necessary for data containing outliers. There is no formal plan to adjust for multiple comparisons. However, multiple correlation analysis will be used to determine the correlation between endpoints, which will aid in assessing the degree of inflation in the overall Type I error rate. This analysis will allow testing of the overall hypothesis that measures of immune function (Objective 1) will be positively correlated with adaptations to exercise (Objective 2) and retention of muscle strength, mass, and function after exercise training is halted (Objective 3). The hypotheses are stated for each objective based on the following lettered time points for comparison:

BASELINE MEASURE TIME POINTS (WEEKS 1 to 5)

A. Blood draw at study enrollment for measuring pre-vaccination antibody titers
B. Assessment of muscle strength, mass, and function at baseline
C. Blood draw prior to acute exercise at baseline
D. Muscle biopsy prior to acute exercise at baseline
E. Blood draw 1 hr after acute exercise at baseline
F. Muscle biopsy 72 hrs after acute exercise at baseline

POST-SUPPLEMENT PRE-TRAINING MEASURE TIME POINTS (WEEK 8)

G. Blood draw prior to acute exercise after weeks of supplementation
H. Muscle biopsy prior to acute exercise after 2 weeks of supplementation
I. Blood draw 1 hr after acute exercise after 2 weeks of supplementation
J. Muscle biopsy 72 hrs after acute exercise after 2 weeks of supplementation

MEASURE TIME POINTS DURING TRAINING PERIOD (WEEKS 9 to 20)

K. Blood draws at weeks 1 and 2 post-vaccination for measuring antibody response

POST-TRAINING MEASURE TIME POINTS (WEEK 21)

L. Assessment of muscle strength, mass, and function after completion of training
M. Muscle biopsy 72 hrs after final exercise bout of training

MEASURES DURING POST-TRAINING FOLLOW-UP (WEEKS 22 to 47)

N. Assessments of strength, mass, and function after 17 and 26 weeks of detraining

#### Objective 1 power and hypotheses

The study will determine if supplement consumption for 2 weeks improves baseline measures of immune function or the immune responses to vaccination or acute exercise. The representative measures chosen to calculate power are change in muscle macrophage content after acute exercise [14] and change in CRP levels after two weeks of supplementation in a randomized double-blind pilot study involving older adults and the current study inclusion and exclusion criteria (unpublished data). Przybyla *et al*. detected a 29% increase in CD163+ macrophages in young subjects after exercise but no change in older subjects [14]. Power for this aim is calculated to detect at least a 29% greater increase in macrophages in the supplement than the placebo group. Przybyla *et al*. used a nonparametric test to lessen the effects of outliers. To be more conservative here, the calculations dropped the most extreme high and low data values before estimating effect sizes. With that approach, the 29% increase corresponds to an effect size of 0.8 SD units (mean/SD = 0.128/0.159). Similar results were found using data suggesting that CRP levels decreases in older adults after supplementation. The effect size for change in CRP is 0.85 SD units (mean/SD = 0.665/0.780 for log-transformed data). Having 20 subjects per group will provide 80% power to detect a mean shift of 0.8 to 0.85 SD units at a significance level of 5% for the following Objective 1 hypotheses:

1. The response to vaccination will be greater in the supplement than the placebo group. The change between times K and A will be compared between groups.
2. The resting ratio of CD4+ to CD8+ t-cells will increase in the supplement but not in the placebo group. The change between times G and C will be compared between groups.
3. The change in t-cell subsets after acute exercise will be greater in the supplement than the placebo group. The change between times I and G will be compared to the change between times E and C for both groups.
4. Resting cytokine levels will decrease in the supplement but not in the placebo group. Change will be compared between groups for blood (times G and C) and muscle (times H and D). Similar results are predicted for the MuRF and MAFbx atrophy-related transcripts.
5. The increase in cytokine levels after acute exercise will be greater in the placebo group than the supplement group. For both groups, change between times I and G will be compared to the change between E and C for blood, and for muscle the change between times J and H will be compared to the change between times F and D.
6. The proliferative capacity of peripheral blood mononuclear cells will be greater in the supplement than the placebo group. The change between times G and C will be compared between groups. The change between times I and E will also be compared between groups.
7. The resting number of muscle CD163+ macrophages will increase in the supplement but not in the placebo group. The change between times H and D will be compared between groups.
8. The number of muscle CD163+ macrophages will increase after acute exercise in the supplement but not in the placebo group. The change between times J and H will be compared to change between times F and D for both groups.
9. Resting NFkB activity will decrease, and PI3K activity will increase in the supplement group but not in the placebo group. The change between time points H and D will be compared between groups.
10. In response to acute exercise, NFkB activity will be lower, and PI3K activity will be greater in the supplement group than in the placebo group. The change between times J and H will be compared to change between times F and D for both groups. Responses similar to NFkB are predicted for FOXO1, and responses similar to PI3K are expected for STAT3, AKT, P38 MAPK, and ERK 1 and 2.

#### Objective 2 power and hypotheses

The study will determine whether supplement consumption increases muscle adaptation to 12 weeks of resistance training. The representative response measure for this objective is post-training percent increase in muscle strength. In a similar study, Trappe *et al*. sought to improve strength gain by resistance training plus drug treatment [26]. The drug treatment group gained significantly more strength by 25.1% than the group receiving placebo (26.4% versus 21.1%, *P* <0.05). Our previous findings indicate that older subjects completing a 12-week training program but not taking supplements will experience an average strength gain of 25 ± 8% [4]. If treatment with the proposed supplement improves strength similarly to the Trappe drug treatment, subjects in the supplement group will improve strength by an average of 31% (that is, 25.1% greater than those of the placebo group). This translates to an approximate effect size of 0.8 SD units (0.25*0.251/0.08) if variances are pooled for the groups compared. With 20 subjects per group, there will be 80% power to detect a mean shift of 0.8 SD units using a significance level of 5% for the Objective 2 hypotheses:

1. The amount of strength gained from resistance training will be greater in the supplement than the placebo group. The change between times L and B will be compared between groups.
2. The amount of muscle size gained from resistance training will be greater in the supplement than the placebo group. The change between times L and B will be compared between groups.
3. The increase in muscle function after resistance training will be greater in the supplement than the placebo group. The change between times L and B will be compared between groups.
4. The increase in muscle satellite cells after resistance training will be greater in the supplement than the placebo group. The change between times M and D will be compared between groups.
5. The increase in muscle myonuclei after resistance training will be greater in the supplement than the placebo group. The change between times M and D will be compared between groups.
6. The increase in muscle fiber size after resistance training will be greater in the supplement group than the placebo group. The change between times M and D will be compared between groups.

#### Objective 3 power and hypotheses

The study will determine if supplement consumption increases the retention of exercise-derived benefits post-training during 26 weeks of long-term follow-up. The representative response measure for this objective is the strength gain retained during long-term follow-up after exercise training is completed. Lemmer *et al*. observed an average gain in muscle strength of 27% (*P* <0.01) in older men after strength training, which is similar to the 25% gain in our study [53]. However, at 31 weeks of post-training follow-up, strength had decreased to be about 9% above the pretraining levels. Assuming the same level of variation among our subjects in retention of strength gain as the observed 8% SD for our study, then a 0.8 SD effect size would be about 6.4% (0.8*0.08), translating to our supplement group retaining strength of 12.4% above pretraining levels. With 20 subjects per group, there will be 80% power to detect a mean shift of 0.8 SD units using a significance level of 5%. If the SD for retention of strength is smaller than 8%, then the study will have higher power to detect the same difference or equal power to detect smaller differences for the following Objective 3 hypotheses:

1. The change in strength during the detraining period will be smaller in the supplement than the placebo group. The change between times N and L will be compared between groups.
2. The change in muscle size during the detraining period will be smaller in the supplement than the placebo group. The change between times N and L will be compared between groups.
3. The change in muscle function will be smaller in the supplement than the placebo group. The change between times N and L will be compared between groups.

## Discussion

The study seeks to determine if a nutritional intervention can target the immune system and complement resistance exercise in helping older adults improve and maintain muscle health. The study is unique in that while exercise is known to have clear effects on immune function [54], little is known about the effects of immune function on individual ability to benefit from exercise. This is because few studies have altered immune function and determined the effects on training outcomes. The proposed study will attempt to do this using a nutritional supplement known to support muscle health. The supplement’s mechanism of action is unknown, but the ingredients alter measures of immune function that will be measured by the study. The study will also examine detraining, an important issue for older adults that is often omitted from training studies [55]. The study will evaluate a nutritional intervention as a complement to exercise for improving muscle health during aging, but the results may only be immediately applicable to relatively healthy older white males due to the stringency of the exclusion criteria and the racial and gender makeup for 60 to 80 year old U.S. veterans. Thus, follow-up studies in other populations, particularly those in need of muscle rehabilitation, may be warranted and necessary. The study also has the potential to advance our understanding of the mechanisms of muscle gain and loss in older adults. Granted, the proposed model (Figure 3) is an ambitious investigation and an oversimplification of the cellular and molecular determinants of muscle phenotype. To overcome this limitation, the study has been approved to allow flexibility in the specific methods and measures of immune function in muscle or blood for practical or scientific reasons and not be considered a protocol deviation so long as the measure 1) addresses a molecular or cellular trait potentially impacted by aging and muscle health or the improvements of muscle health by supplementation or exercise; 2) is not genetic testing; and 3) the results will not be communicated back to the subjects or used to influence their medical care in any way.

In conclusion, a randomized controlled trial of nutritional supplement use by older adults during resistance exercise training and post-training follow-up will be conducted. The individual benefits and potential scientific advancement far outweigh the risks of participation. High intensity training can be safely performed with benefit by older adults including nursing home residents over 90 years of age [56]. However, the magnitude of the response to training varies significantly even between young and healthy individuals [3]. Considering this interindividual variation, complementary interventions such as nutritional supplementation and a better understanding of the role of immune function in muscle adaptation to training and detraining may be valuable in optimizing preventative and rehabilitative exercise programs for older adults.

Thus, the importance of the scientific knowledge to be gained is also high. The risks of exercise, the supplement ingredients, and percutaneous muscle biopsy are relatively low compared to the possibility that targeting the immune system may be the advantage needed for certain older adults to successfully maintain or restore the mass, strength, and function that is necessary for personal independence, health, and quality of life [57-59].

## Trial status

The study has been active and open for recruitment since 5 August 2014.

## Abbreviations

ARG: arginine
GLN: glutamine
HMB: β-Hydroxy-β-methylbutyrate
RM: repetition maximum
TDAP: tetanus, diphtheria, and pertussis
VA: Veterans Affairs
